# Association of Dexmedetomidine With Postoperative Depressive Symptoms in Older Surgical Patients: A Prospective Multicenter Study

**DOI:** 10.1111/cns.70407

**Published:** 2025-05-19

**Authors:** Xinyu Hao, Zhuoning Zhang, Lujia Yang, Yongxin Guo, Fuyang Cao, Jiangbei Cao, Yanhong Liu, Jingsheng Lou, Ziyao Xu, Yulong Cui, Yunxiao Bai, Xiaoping Gu, Difen Wang, Qianyu Cui, Zhikang Zhou, Hao Shen, Jingjia Sun, Weidong Mi, Li Tong

**Affiliations:** ^1^ Department of Anesthesiology The First Medical Centre, Chinese PLA General Hospital Beijing China; ^2^ Department of Anesthesiology The Sixth Medical Centre, Chinese PLA General Hospital Beijing China; ^3^ Department of General Surgery The First Medical Centre, Chinese PLA General Hospital Beijing China; ^4^ Department of Anesthesiology The Second Xiangya Hospital of Central South University Changsha China; ^5^ Department of Anesthesiology, Union Hospital, Tongji Medical College Huazhong University of Science and Technology Wuhan China; ^6^ Department of Anesthesiology Drum Tower Clinical Medical College of Nanjing Medical University Nanjing Jiangsu China; ^7^ Department of Critical Care Medicine The Affiliated Hospital of Guizhou Medical University Guiyang Guizhou China; ^8^ Department of Anesthesiology, Beijing Tiantan Hospital Capital Medical University Beijing China; ^9^ National Clinical Research Center for Geriatric Diseases The Second Medical Center, Chinese PLA General Hospital Beijing China

**Keywords:** anxiety symptom, delirium, depressive symptom, dexmedetomidine, noncardiac surgery, older patients

## Abstract

**Background:**

Neuropsychiatric symptoms significantly impact surgical recovery, quality of life, and long‐term survival. To investigate the association between intraoperative dexmedetomidine administration and the incidence of postoperative depressive symptoms in noncardiac surgical patients.

**Methods:**

A multicenter prospective observational study of older surgical patients over 65 years of age from April 2020 to April 2022. The primary outcome was the incidence of postoperative 7‐day depressive symptoms. Secondary outcomes were the incidence of postoperative 7‐day anxiety symptoms, sleep disturbance, and delirium. A logistic regression model based on the random effect was used to determine the association between dexmedetomidine administration and the outcomes. Propensity score matching (PSM) and inverse probability treatment weighting (IPTW) were employed to address data imbalance. Subgroup analyses based on specific populations were performed to explore the relationship between dexmedetomidine and depressive symptoms.

**Results:**

Of 5591 patients, 20.5% (1148) received intraoperative dexmedetomidine. The incidence of postoperative 7‐day depressive symptoms was significantly lower in the dexmedetomidine group compared to the nondexmedetomidine group (unadjusted: 7.6% vs. 26.7%, *p* < 0.001; PSM: 7.9% vs. 29.0%, IPTW: 8.7% vs. 25.8%, *p* < 0.001). Dexmedetomidine was significantly associated with the remission of postoperative 7‐day depressive symptoms (adjusted random‐effect model: risk ratio [RR] 0.104, 95% CI, 0.080–0.140, *p* < 0.001; PSM: RR 0.311, 95% CI, 0.242–0.415, *p* < 0.001; IPTW: RR 0.297, 95% CI, 0.253–0.343, *p* < 0.001). Additionally, dexmedetomidine demonstrated protective effects against postoperative anxiety symptoms, sleep disturbance, and delirium. In age, gender, cumulative comorbidity, frailty, ASA physical status, and inhaled anesthetic subgroups, we also found that dexmedetomidine was associated with a reduction in postoperative depressive symptoms in older noncardiac patients.

**Conclusion:**

Intraoperative dexmedetomidine administration was associated with a reduction in postoperative 7‐day depressive symptoms, anxiety symptoms, sleep disturbances, and delirium in older patients undergoing noncardiac surgery.

**Trial Registration:**

The clinical trial protocol of this study was registered in the Clinical Trial registry (NCT06362408).

**Prior Presentations:**

The authors have nothing to report.

## Graphical abstract

This multicenter prospective observational study was conducted in China from April 2020 to April 2022, focusing on elderly surgical patients aged 65 years and older who underwent noncardiac surgery. The study revealed that the use of dexmedetomidine during the intraoperative phase significantly diminished the incidence of depressive and anxiety symptoms, sleep disturbances, and delirium within the first 7 days postoperatively. Through the application of adjusted random‐effects logistic regression analysis, propensity score matching (PSM), and inverse probability treatment weighting (IPTW) techniques, it was conclusively established that the administration of dexmedetomidine during surgery was robustly linked to a marked decrease in the manifestation of these postoperative neuropsychiatric symptoms at the 7‐day mark. Dexmedetomidine presents itself as a potentially groundbreaking approach and direction for the management of postoperative neuropsychiatric complications in elderly surgical patients. As a result, there is a compelling need for further randomized controlled trials to substantiate the therapeutic benefits of dexmedetomidine in mitigating postoperative neuropsychiatric symptoms in older adults undergoing noncardiac surgical procedures.

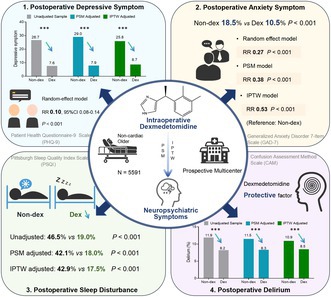

## 1 Introduction

Postoperative depressive symptoms represent one of the neuropsychiatric complications that can manifest as a depressed mood, loss of interest, difficulty concentrating, and a diminished sense of self‐worth following surgery [1]. Generally, the incidence of postoperative depressive symptoms in the surgical population ranges from 10% to 30% [2]. Older patients are at a higher risk for depressive symptoms due to various factors, including physical, psychological, and social support issues [3]. Some studies indicate that the incidence of postoperative depressive symptoms in older patients may be as high as 30%–50% [4].

Managing postoperative depressive symptoms remains a significant clinical challenge. On the one hand, depressive symptoms can hinder older patients' engagement in the recovery process, thereby increasing the risk of postoperative complications [5]. On the other hand, these negative emotional issues can diminish life satisfaction, impact future life and work capabilities, and may lead patients to seek medical assistance more frequently, ultimately increasing healthcare costs [6]. While pharmacological treatments such as antidepressants and anxiolytics are commonly used in clinical settings, their efficacy and safety in older patients are often compromised by side effects and drug interactions. Consequently, there is a growing body of research focused on identifying perioperative alternative strategies to alleviate negative neuropsychiatric symptoms that arise postoperatively without introducing additional risks [7].

Dexmedetomidine, an alpha‐2‐adrenergic receptor agonist, was initially utilized in the intensive care setting primarily for sedation and analgesia in critically ill patients [8]. Due to its less respiratory depressive symptoms, the advantages of reduced demand for anesthetic drugs and flexible delivery methods have been rapidly promoted to other clinical application scenarios [9]. For instance, Dong et al. found that nocturnal administration of dexmedetomidine reduced the incidence of intensive care unit syndrome in cardiac surgery patients [10]. In 2022, a report in *JAMA* by Sheldon et al. indicated that sublingual dexmedetomidine effectively alleviated agitation in patients with bipolar disorder [11]. Additionally, a clinical trial by Su et al. demonstrated that low‐dose dexmedetomidine could decrease the occurrence of delirium in older patients undergoing noncardiac surgery [12]. However, Turan et al. reported that dexmedetomidine did not significantly reduce the incidence of postoperative delirium or atrial fibrillation in patients undergoing cardiac surgery [13]. Moreover, preclinical trials have shown that dexmedetomidine can mitigate anxiety symptom‐like behavior following peripheral nerve injury in mice by reducing the hyperactivity of glutamatergic neurons [14]. In a randomized controlled trial involving 310 emergency patients, dexmedetomidine was found to alleviate anxiety symptoms in those experiencing traumatic stress [15]. Previous studies have highlighted the overlap between anxiety symptoms and depressive symptoms, indicating that comorbidities may occur [16]. Notably, while available clinical guidelines and studies have well‐documented the role of dexmedetomidine in relieving anxiety symptoms, fewer studies have explored its relationship with depressive symptoms. A recent randomized controlled trial reported that a single dose of dexmedetomidine reduced postpartum depressive symptoms in women, but its effects on other patients undergoing noncardiac surgery remain unclear [17].

To our knowledge, there have been few studies to date that examine the association between dexmedetomidine and postoperative neuropsychiatric complications, such as depressive symptoms, in patients over 65 years of age who have undergone noncardiac surgery. Therefore, we utilized prospective observational data from nine multicenter general hospitals in China to investigate the relationship between intraoperative dexmedetomidine administration and the occurrence of postoperative depressive symptoms, anxiety symptoms, sleep disturbance, and delirium in older patients undergoing noncardiac surgery.

## 2 Methods

### 2.1 Study Design

This cohort analysis was a sub‐study of the Perioperative Database of Chinese Elderly Patients (PDCEP) study. We conducted a statistical analysis on data from older patients aged 65 years and above who underwent noncardiac surgery across nine general hospitals in China between April 1, 2020, and April 30, 2022. The multicenter prospective observational data were collected from nine comprehensive tertiary hospitals located in Beijing (four hospitals), Guangxi (one hospital), Guizhou (one hospital), Hunan (two hospitals), and Jiangsu (one hospital) provinces. The study was conducted in accordance with the Declaration of Helsinki and received approval from the Ethics Committee Board of the First Medical Center of the Chinese PLA General Hospital (S2024‐024‐03). Additionally, it was registered on Clinical Trials.gov (NCT06362408). The manuscript adheres to the applicable Strengthening the Reporting of Observational Studies in Epidemiology (STROBE) guidelines.

### 2.2 Participant Selection

This study included older patients undergoing a variety of noncardiac surgical procedures, encompassing otolaryngology, urology, gynecology, gastroenterology, orthopedics, thoracic surgery, and hepatobiliary surgery. Eligible patients were divided into two groups: the dexmedetomidine group and the nondexmedetomidine group. Patients who received dexmedetomidine (administered within the therapeutic range of 0.2–1.0 μg/(kg·h)) for anesthesia induction or maintenance during the surgical procedures were assigned to the dexmedetomidine group. Conversely, those who did not receive dexmedetomidine at any stage of the anesthesia process were classified into the nondexmedetomidine group.

Inclusion criteria were as follows: (a) patients aged 65 years or older who underwent elective noncardiac surgeries, (b) American Society of Anesthesiologists (ASA) physical status I–III, (c) administration of general anesthesia (such as intravenous compound anesthesia or intravenous general anesthesia), and (d) surgical duration exceeding 60 min. Exclusion criteria were as follows: (a) patients with preexisting psychiatric conditions (such as major depressive disorder, anxiety symptom disorder, or schizophrenia) or those currently taking medications that affect mental status (including antidepressants, anxiolytics, or sedatives), (b) previous long‐term sleep disturbance or regular use of sleeping pills, (c) patients with poor postoperative recovery, prolonged hospitalization, or those requiring intensive care, in order to avoid confounding the outcomes of postoperative evaluations, as well as death within 7 days following surgery, (d) missing data regarding the exposure factor, and (e) patients lacking sufficient language skills to understand the study content or to participate in the assessment, as well as those with absent outcome data.

### 2.3 Data Collection and Extraction

Based on the current situation of data application in each center and the requirement of data security control, a targeted processing method was adopted, including File Transfer Protocol as a data source in large clinical data centers. The data were synchronized in quasi‐real time by means of a Web‐Service data service interface. The data were exported through the subcenter locally established perioperative database. Data backup was carried out through the business system, and data files were transferred to the main database by means of a network terminal or mobile media copy. The following relevant study variables are involved in this study:

1. We extracted demographic information about the patients' age, gender, BMI, education level, smoking, and alcohol history.
2. Preoperative indicators include the patient's history of previous surgery, history of chronic pain, history of malignancy, cumulative comorbidities (diabetes mellitus, congestive heart failure, secondary hypertension, chronic bronchitis, chronic obstructive pulmonary disease, asthma, stroke, hepatitis, and kidney disease), frailty state, and metabolic equivalents. The last laboratory test before surgery includes white blood cell count and hemoglobin.
3. Intraoperative indicators in older patients involved the American Society of Anesthesiologists (ASA) physical status, surgical grade, use of inhalation anesthetics, use of nonsteroidal drugs, use of colloids, and allogeneic blood transfusion.
4. Postoperative variables include postoperative drainage and postoperative analgesia.

### 2.4 Outcomes Measure

Primary outcome was the incidence of postoperative within 7‐day depressive symptoms. The Patient Health Questionnaire‐9 (PHQ‐9) was a validated tool designed to screen for depressive symptoms based on the nine diagnostic criteria for major depressive disorder outlined in the Diagnostic and Statistical Manual of Mental Disorder (DSM‐IV) [18]. Patients rate the frequency of symptoms experienced over the past 7 days on a 4‐point Likert scale (0 = “not at all” to 3 = “nearly every day”), yielding a total score ranging from 0 to 27, with higher scores indicating greater severity [19]. Standard cutoff values classify depressive symptom severity as mild (5–9), moderate (10–15), and severe (16–27) [20]. In this study, a 5‐point criterion was used to define whether or not there was a positive depressive symptoms.

Secondary outcomes were the incidence of postoperative within 7‐day anxiety symptoms, sleep disturbance, and delirium symptoms. The Generalized Anxiety Symptoms Disorder‐7 (GAD‐7) was a validated, self‐reported questionnaire used to assess the severity of anxiety symptoms. It consists of seven items that align with the diagnostic criteria for generalized anxiety symptom disorder as outlined in the DSM‐IV [21]. The total score ranges from 0 to 21, with higher scores indicating more severe anxiety symptoms. Standard cutoff values are used to classify symptom severity: mild (5–9), moderate (10–15), and severe (16–21). In this study, the GAD‐7 was employed as a binary variable (positive screening for anxiety symptoms with a score ≥ 5) [22]. Trained personnel administered the GAD‐7 and were instructed to provide clear and unbiased guidance while maintaining confidentiality. For patients who screened positive on the assessment scales, an evaluation by a qualified psychiatrist was conducted to confirm the presence of clinical symptoms or establish a definitive diagnosis. Moreover, sleep disturbance was identified using the Pittsburgh Sleep Quality Index (PSQI), a widely validated tool for assessing subjective sleep quality and disturbances after surgery [23]. The PSQI consists of 19 self‐reported items grouped into seven components: subjective sleep quality, sleep latency, sleep duration, habitual sleep efficiency, sleep disturbances, use of sleeping medications, and daytime dysfunction [24]. Each component is scored on a scale of 0–3, with a total score ranging from 0 to 21. A score of 0–10 indicates normal sleep quality, a score of 11–15 indicates average sleep quality, and a score of 16–21 indicates sleep disturbance [25]. A higher PSQI score indicates poorer sleep quality and a total score > 8 was commonly used as the cutoff for identifying significant sleep disturbances. In addition, delirium was assessed in older patients using the 3‐min Diagnostic Interview for Confusion Assessment Method (3D‐CAM) [26]. Assessments are performed twice a day for 7 consecutive days or until discharge. Morning and evening assessments were conducted at 10:00 a.m. and 4:00 p.m. If the delirium was positive at any of the follow‐up visits, we can consider the postoperative delirium positive. This study selected validated standardized scales that have good reliability and validity in different populations.

Adopted the following methods to ensure the quality and standardization of scale data: first, provide systematic training to all follow‐up staff and evaluators to familiarize them with the scale's structure, scoring rules, and key definitions, ensuring that they were able to assess in a consistent manner. Secondly, interrater consistency tests (such as kappa coefficient) are regularly performed to monitor scoring agreement between different raters. Furthermore, follow‐up staff must communicate with patients in a clear, polite, and unbiased manner, avoiding suggestive questions. It is essential to maintain a neutral attitude, ensuring that the patient is not influenced by external factors while responding to questions. The assessment will be conducted through face‐to‐face interviews. If a patient opts for discharge within 7 days, follow‐up will be facilitated via online video conferencing or telephone consultations.

### 2.5 PSM and IVTW Analysis

Propensity score matching (PSM) was employed to align experimental and control groups by estimating the probability of subjects receiving specific treatments, thereby mitigating the effects of selection bias and enhancing the assessment of treatments' outcomes on study results [27]. Following propensity score calculation, patients categorized into dexmedetomidine and nondexmedetomidine groups were matched in a 1:1 ratio through the greedy nearest neighbor matching technique, with a maximum caliper width of 0.2. Standardized mean differences (SMD) were utilized to assess the equilibrium among group variables, where SMD below 0.2 suggested adequate balance. Inverse probability of treatment weighting (IPTW) was implemented by assigning weights based on the inverse of the sample selection probability, aiming to more precisely quantify the relationship between exposure variables and research outcomes [28]. Propensity score kernel density plots were employed to evaluate the equivalence between matched patients.

### 2.6 Sample Size Calculation

Previous studies have reported a 30% incidence of postoperative depressive symptoms in elderly patients undergoing noncardiac surgery. Based on previous studies of dexmedetomidine, we conservatively estimate a 6% reduction in the incidence of postoperative depressive symptoms in the dexmedetomidine group. We hypothesized that the incidence of postoperative depressive symptoms in older patients undergoing noncardiac surgery was 24% in the dexmedetomidine group and 30% in the patients nondexmedetomidine group, with a risk ratio of 1.25 (30%/24%). To obtain 80% power and an alpha value of 0.05, we calculated that 859 patients were needed per group. Considering the prospective acceptable loss to follow‐up rate of 10%, 1890 cases (945 cases in each group) were finally included. Sample size calculations were performed using the PASS (version 15) software.

### 2.7 Statistical Analysis

A normality test was performed for continuous variables. The skewed continuous data were presented as median and interquartile ranges, and the differences between groups were compared by Mann–Whitney U‐test. Categorical variables were presented in terms of numbers and percentages and analyzed using either the χ^2^ test or Fisher's exact test, as appropriate. Patient demographic data (age, gender, Minimum Mental State Examination scores [MMSE], body mass index, education level, smoking, and alcohol consumption), preoperative variables (history of surgery, chronic pain, history of malignancy, cumulative comorbidity, frailty state, metabolic equivalent [MET], leukocytes, and hemoglobin), and surgical and anesthetic information (American Society of Anesthesiologists [ASA] physical status classification, duration of surgery, surgical grade, use of inhaled anesthesia, use of nonsteroidal anti‐inflammatory drugs [NSAIDs], colloidal use and blood transfusion, postoperative drains, patient‐controlled analgesia, type of surgery, and follow‐up method) were adjusted in all models. We used random‐effects logistic analysis to analyze the association of intraoperative dexmedetomidine with neuropsychiatric symptoms in older patients undergoing noncardiac surgery. To determine whether the source of heterogeneity between different subgroups affects the association between dexmedetomidine and the outcomes, we used forest plots to summarize the association between subgroups of different ages (65 ~ 75 years vs. > 75 years), gender (male vs. female), cumulative comorbidity (< 2 vs. ≥ 2), frailty status (robust vs. prefrail vs. frail), ASA physical status (I/II vs. III), and use of inhaled anesthetics (no vs. yes) with postoperative psychological symptoms. Statistical analyses were conducted using IBM SPSS Statistics version 26.0, GraphPad Prism version 8.0, and R Statistical software. Statistical significance was determined at a two‐tailed *p* value threshold of less than 0.05.

## 3 Result

### 3.1 Patient Selection and Characteristics

**FIGURE 1:**
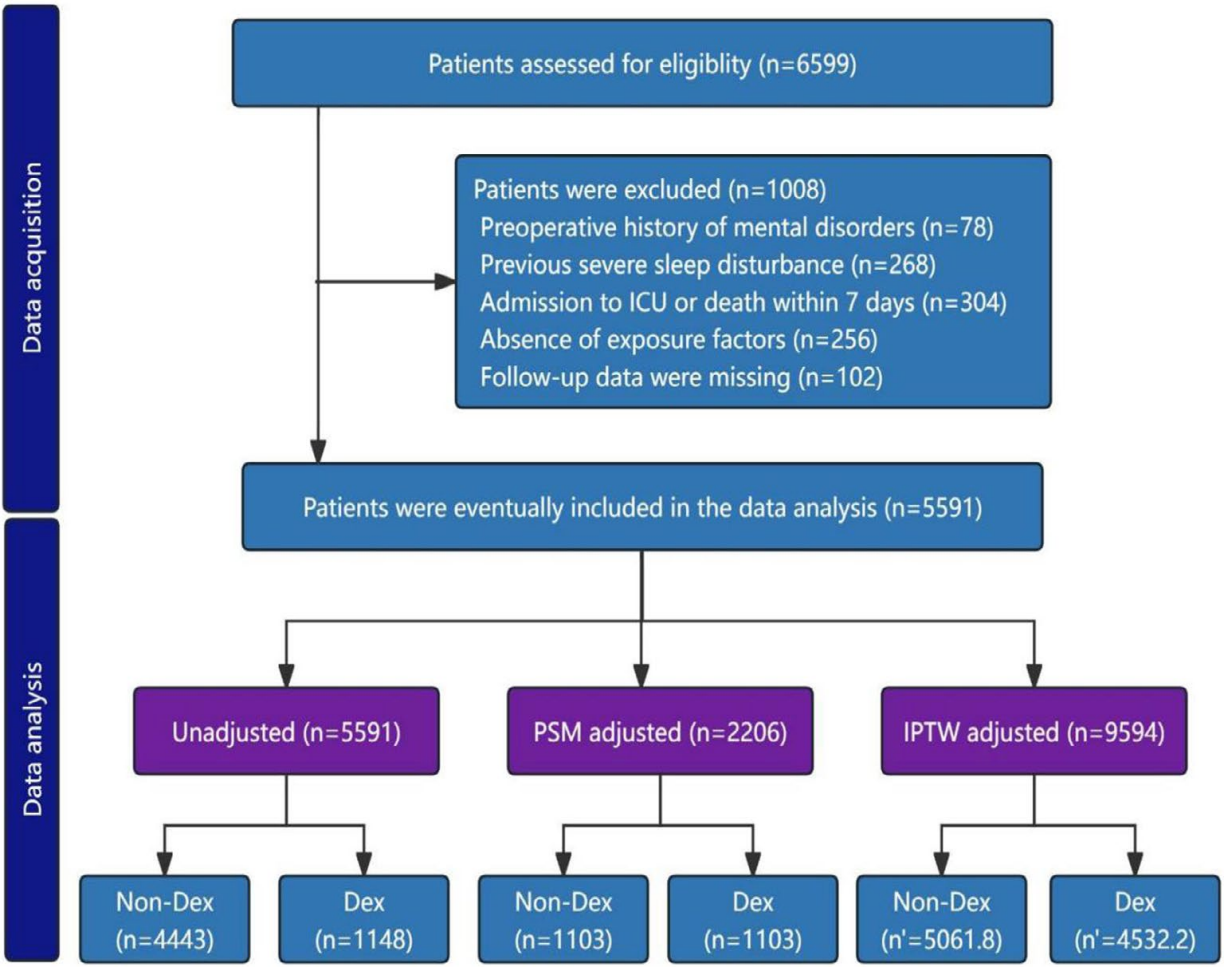
Flowchart of the study.

**FIGURE 2:**
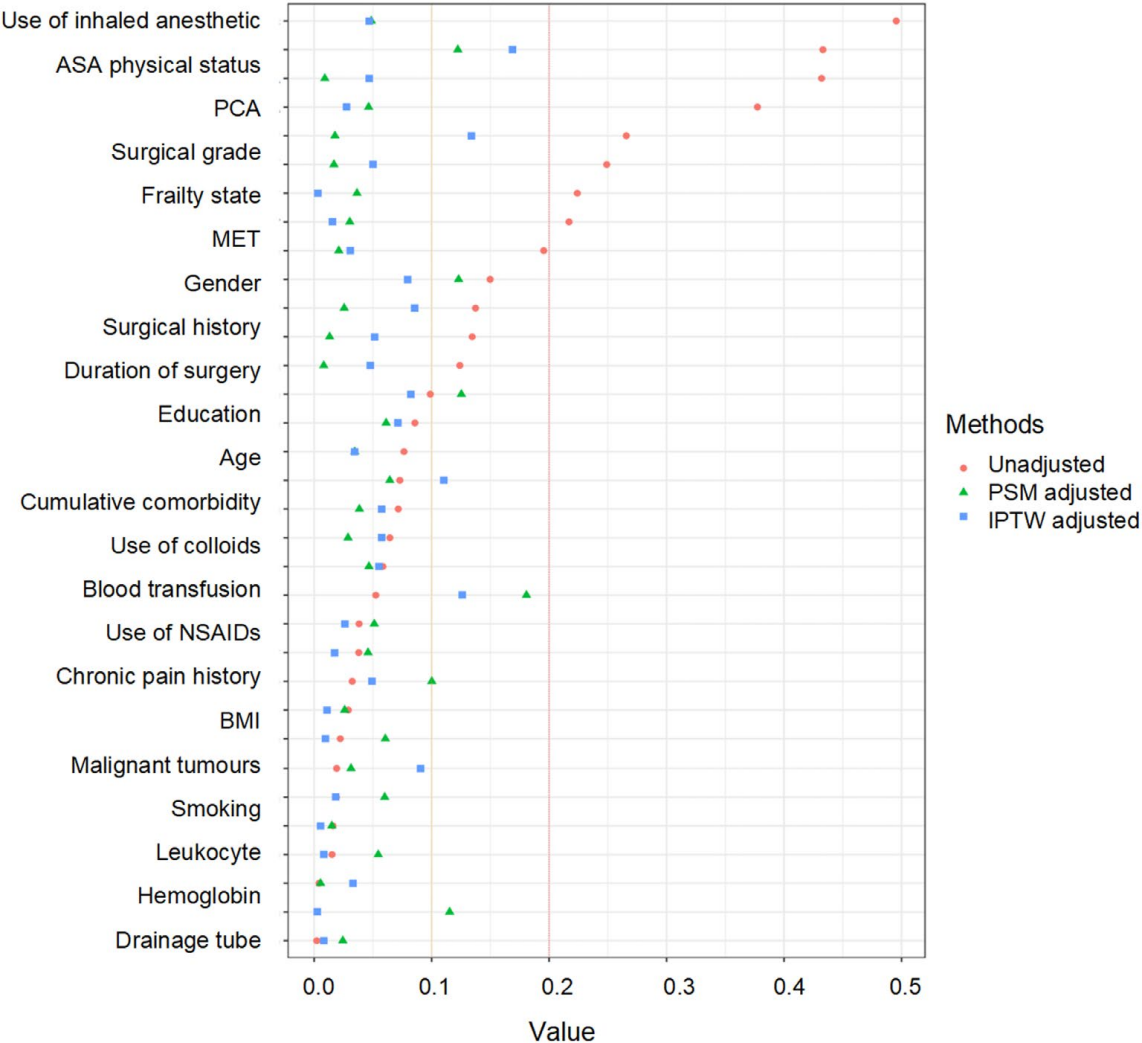
The standardized mean difference of variables before and after PSM and IPTW adjustments. ASA, American Society of Anesthesiologists; BMI, body mass index; COPD, chronic obstructive pulmonary disease; IPTW, inverse probability of treatment weighting; MET, metabolic equivalent; NSAID, nonsteroidal anti‐inflammatory drugs; PCA, patient‐controlled analgesia; PSM, propensity score matching; SMD, standard mean difference.

A total of 6599 older patients aged 65 years or older who underwent noncardiac surgery were screened, and 5591 patients were included in the analysis after exclusion criteria (Figure 1). After 1:1 PSM adjustment, the dexmedetomidine and nondexmedetomidine groups each contained 1103 patients. Following IPTW adjustment, the DEX and nondexmedetomidine groups were composed of 5061.8 and 4532.2 patients, respectively. Figure S1 showed the use of dexmedetomidine in seven noncardiac surgery departments, of which 36.8% were used for urological surgery, 19.8% for gastrointestinal surgery, and 3.7% for gynecological surgery. A kernel density plot illustrates the transition of data from a scattered to a matched and well‐fitted distribution postmatching (Figure S2). 20.5% (*n* = 1148/5591) of patients received dexmedetomidine management, with a median age of 69 years, 66.5% were male, 48.7% had a prior surgical history, and 20.2% presented with ≥ 2 comorbidities (Table 1). After PSM adjustment, the dexmedetomidine and nondexmedetomidine groups each consisted of 1103 patients, with a median age of 69 years in the dexmedetomidine group and 70 years in the nondexmedetomidine group. After IPTW adjustment, the median age in both the dexmedetomidine and nondexmedetomidine groups was 70 years. In the unadjusted dataset, significant imbalances were observed in gender, history of chronic pain, hypertension, stroke, hepatitis, frailty, ASA physical status, use of inhaled anesthetics, and postoperative analgesia (SMD > 0.2). The SMD of the variables shown in Figure 2 was less than 0.2 after the PSM and IPTW adjustment, indicating that the data distribution was uniformly balanced.

**TABLE 1:** Demographic and perioperative data characteristics of unadjusted and adjusted samples.

Characteristics	Unadjusted sample (*n* = 5591)				PSM adjusted (*n* = 2206)			IPTW adjusted (*n* = 9594)		
	Non‐DEX (*n* = 4443)	DEX (*n* = 1148)	*p*	SMD	Non‐DEX (*n* = 1103)	DEX (*n* = 1103)	SMD	Non‐DEX (*n’* = 5061.8)	DEX (*n’* = 4532.2)	SMD
Demographic characteristics										
Age, years, median (IQR)	70 (67, 74)	69 (66, 74)	< 0.001	0.099	70 (67, 74)	69 (66, 74)	0.125	70 (67, 74)	70 (66, 74)	0.082
Gender, male, *n* (%)	2486 (56.0)	763 (66.5)	< 0.001	0.217	703 (63.7)	719 (65.2)	0.030	3030.8 (59.9)	2748.1 (60.6)	0.016
MMSE score, median (IQR)	27 (26, 29)	27 (26, 29)	0.673	0.078	27 (26, 28)	27 (26, 28)	0.012	27 (26, 28)	27 (26, 28)	0.023
BMI, kg/m^2^, *n* (%)			0.520	0.038			0.051			0.026
< 18.5	112 (2.5)	24 (2.1)			28 (2.5)	24 (2.2)		117.6 (2.3)	97.8 (2.2)	
18.5–25	3640 (81.9)	934 (81.4)			878 (79.6)	900 (81.6)		4107.3 (81.1)	3643.6 (80.4)	
> 25	691 (15.6)	190 (16.6)			197 (17.9)	179 (16.2)		836.9 (16.5)	790.8 (17.4)	
Education level, *n* (%)			< 0.001	0.124			0.008			0.048
< 9 years	3436 (77.3)	826 (72.0)			807 (73.2)	803 (72.8)		3783.3 (74.7)	3291.9 (72.6)	
≥ 9 years	1007 (22.7)	322 (28.0)			296 (26.8)	300 (27.2)		1278.6 (25.3)	1240.3 (27.4)	
Smoking, *n* (%)	1181 (26.6)	320 (27.9)	0.399	0.029	326 (29.6)	313 (28.4)	0.026	1403.3 (27.7)	1234.7 (27.2)	0.011
Alcohol consumption, *n* (%)	1121 (25.2)	261 (22.7)	0.087	0.058	277 (25.1)	255 (23.1)	0.047	1320.6 (26.1)	1074.7 (23.7)	0.055
Preoperative variables										
Surgical history, *n* (%)	1595 (35.9)	538 (46.9)	< 0.001	0.001	486 (44.1)	506 (45.9)	0.036	2006.1 (39.6)	1789.5 (39.5)	0.003
Chronic pain history, *n* (%)	440 (9.9)	101 (8.8)	0.283	0.259	115 (10.4)	100 (9.1)	0.046	508.3 (10.0)	431.6 (9.5)	0.017
Malignant tumor history, *n* (%)	1796 (40.4)	240 (20.9)	< 0.001	0.001	185 (16.8)	238 (21.6)	0.038	1603.6 (31.7)	1093.5 (24.1)	0.169
Comorbidity, *n* (%)										
Diabetes mellitus	910 (20.5)	206 (17.9)	0.061	0.055	184 (16.7)	196 (17.8)	0.029	984.5 (19.4)	780.6 (17.2)	0.058
Coronary heart disease	710 (16.0)	109 (9.5)	< 0.001	< 0.001	184 (16.7)	196 (17.8)	0.021	583.3 (11.5)	478.3 (10.6)	0.031
Hypertension	2088 (47.0)	538 (46.9)	0.963	0.937	451 (40.9)	514 (46.6)	0.115	2308.6 (45.6)	2073.3 (45.7)	0.003
Chronic bronchitis	117 (2.6)	31 (2.7)	0.982	0.004	32 (2.9)	31 (2.8)	0.012	141.0 (2.8)	152.1 (3.4)	0.033
COPD	92 (2.1)	27 (2.4)	0.636	0.019	21 (1.9)	26 (2.4)	0.046	99.0 (2.0)	155.1 (3.4)	0.091
Asthma	35 (0.8)	3 (0.3)	0.083	0.073	8 (0.7)	3 (0.3)	0.061	39.9 (0.8)	3.1 (0.1)	0.111
Stroke	153 (3.4)	40 (3.5)	0.999	0.946	42 (3.8)	37 (3.4)	0.009	175.6 (3.5)	164.2 (3.6)	0.008
Hepatitis	160 (3.6)	38 (3.3)	0.699	0.634	39 (3.5)	36 (3.3)	0.015	171.5 (3.4)	149.1 (3.3)	0.005
Kidney disease	185 (4.2)	30 (2.6)	0.019	0.015	42 (3.8)	30 (2.7)	0.061	198.1 (3.9)	119.8 (2.6)	0.071
Cumulative comorbidity, *n* (%)			< 0.001	0.001			0.013			0.052
< 2	3210 (72.2)	896 (78.0)			865 (78.4)	859 (77.9)		3816.7 (75.4)	3516.5 (77.6)	
≥ 2	1233 (27.8)	252 (22.0)			238 (21.6)	244 (22.1)		1245.1 (24.6)	1015.7 (22.4)	
Frailty state, *n* (%)			< 0.001	0.266			0.018			0.134
Robust	3727 (83.9)	1051 (91.5)			1005 (91.1)	1006 (91.2)		4522.4 (89.3)	4104.6 (90.6)	
Prefrail	521 (11.7)	86 (7.5)			85 (7.7)	86 (7.8)		472.5 (9.3)	419.7 (9.3)	
Frail	195 (4.4)	11 (1.0)			13 (1.2)	11 (1.0)		67.0 (1.3)	7.8 (0.2)	
MET, *n* (%)			< 0.001	0.249			0.017			0.05
10	617 (13.9)	231 (20.1)			231 (20.9)	224 (20.3)		840.9 (16.6)	789.9 (17.4)	
5 ~ 9	2512 (56.5)	512 (44.6)			503 (45.6)	510 (46.2)		2686.2 (53.1)	2291.3 (50.6)	
0 ~ 4	1314 (29.6)	405 (35.3)			369 (33.5)	369 (33.5)		1534.7 (30.3)	1451.0 (32.0)	
Leukocyte, 10^9^/L, median (IQR)	6 (5, 7)	6 (5, 8)	< 0.001	0.022	6 (5, 8)	6 (5, 8)	0.061	6 (5, 7)	6 (5, 8)	0.01
Hemoglobin, g/dl, *n* (%)			0.610	0.019			0.006			0.019
≥ 10	4020 (90.5)	1045 (91.0)			981 (88.9)	1001 (90.8)		4570.9 (90.3)	4117.2 (90.8)	
< 10	423 (9.5)	103 (9.0)			122 (11.1)	102 (9.2)		491.0 (9.7)	415.0 (9.2)	
Intraoperative variables										
ASA classification, *n* (%)			< 0.001	0.432			0.009			0.047
I/II	3118 (70.2)	568 (49.5)			563 (51.0)	568 (51.5)		3228.3 (63.8)	2787.9 (61.5)	
III	1325 (29.8)	580 (50.5)			540 (49.0)	535 (48.5)		1833.5 (36.2)	1744.2 (38.5)	
Duration of surgery, h, *n* (%)			< 0.001	0.137			0.026			0.085
< 3	2712 (61.0)	623 (54.3)			607 (55.0)	621 (56.3)		3075.2 (60.8)	2562.9 (56.5)	
≥ 3	1731 (39.0)	525 (45.7)			496 (45.0)	482 (43.7)		1986.7 (39.2)	1969.3 (43.5)	
Surgical grade, *n* (%)			0.621	0.032			0.099			0.049
1/2	424 (9.5)	104 (9.1)			119 (10.8)	103 (9.3)		516.1 (10.2)	432.9 (9.6)	
3	1099 (24.7)	272 (23.7)			308 (27.9)	271 (24.6)		1347.3 (26.6)	1128.8 (24.9)	
4	2920 (65.8)	772 (67.2)			676 (61.3)	729 (66.1)		3198.5 (63.2)	2970.5 (65.5)	
Use of inhaled anesthetic, *n* (%)	4027 (90.6)	825 (71.9)	< 0.001	0.496	846 (76.7)	823 (74.6)	0.049	4317.6 (85.3)	3788.5 (83.6)	0.047
Use of NSAIDs, *n* (%)	1843 (41.5)	506 (44.1)	0.120	0.052	386 (35.0)	483 (43.8)	0.181	1948.4 (38.5)	2025.8 (44.7)	0.126
Use of colloids, *n* (%)	2296 (51.7)	637 (55.5)	0.023	0.076	587 (53.2)	606 (54.9)	0.035	2657.9 (52.5)	2457.9 (54.2)	0.035
Blood transfusion, *n* (%)	710 (16.0)	151 (13.2)	0.038	0.08	169 (15.3)	154 (14.0)	0.038	783.0 (15.5)	609.5 (13.4)	0.057
Drainage tube, *n* (%)	3154 (71.0)	807 (70.3)	0.672	0.015	739 (67.0)	767 (69.5)	0.055	2109.3 (41.7)	1826.8 (40.3)	0.028
PCA, *n* (%)	2156 (48.5)	349 (30.4)	< 0.001	0.377	373 (33.8)	349 (31.6)	0.046	4317.6 (85.3)	3788.5 (83.6)	0.047

*Note:* Data were presented as median (IQR) or *n* (%).

Abbreviations: ASA, American Society of Anesthesiologists; BMI, body mass index; COPD, chronic obstructive pulmonary disease; DEX, dexmedetomidine; IPTW, inverse probability of treatment weighting; MET, metabolic equivalent; NSAID, nonsteroidal anti‐inflammatory drugs; PCA, patient‐controlled analgesia; PSM, propensity score matching; SMD, standard mean difference.

### 3.2 Primary Outcome Analysis

We found that the incidence of postoperative depressive symptoms was significantly lower in intraoperative dexmedetomidine patients than in nondexmedetomidine patients (unadjusted: 7.6% vs. 26.7%, *p* < 0.001; PSM adjusted: 7.9% vs. 29.0%, *p* < 0.001; IPTW adjusted: 8.7% vs. 25.8%, *p* < 0.001, Table 2). Patients with mild depressive symptoms accounted for the highest proportion, and patients with severe symptoms accounted for the lowest. It was worth noting that after adjustment, dexmedetomidine was significantly lower than that of the nondexmedetomidine group only in the comparison of mild and moderate depressive symptoms, and there was no significant difference in severe depressive symptoms (PSM adjusted mild: 4.5% vs. 19.9%, *p* < 0.001; PSM adjusted moderate: 1.8% vs. 5.3%, *p* = 0.002; IPTW adjusted mild: 4.5% vs. 18.1%, *p* < 0.001; IPTW adjusted moderate: 2.1% vs. 4.8%, *p* = 0.002). The incidence of postoperative depressive symptoms in the dexmedetomidine group was significantly lower than that in the nondexmedetomidine group in patients undergoing otolaryngology, urology, gynecology, orthopedics, gastrointestinal surgery, hepatobiliary surgery, and thoracic surgery (Figure S3).

**TABLE 2:** Compare the differences in primary and secondary outcomes of the data before and after the PSM and IPTW adjustments.

Characteristics	Unadjusted sample (*n* = 5591)			PSM adjusted (*n* = 2206)			IPTW adjusted (*n* = 9594)		
	Non‐DEX (*n* = 4443)	DEX (*n* = 1148)	*p*	Non‐DEX (*n* = 1103)	DEX (*n* = 1103)	*p*	Non‐DEX (*n’* = 5061.8)	DEX (*n’* = 4532.2)	*p*
Primary outcome									
Depressive symptoms, *n* (%)	1186 (26.7)	87 (7.6)	< 0.001	320 (29.0)	87 (7.9)	< 0.001	1303.9 (25.8)	394.0 (8.7)	< 0.001
Severity of depressive symptoms, *n* (%)									
Mild	852 (19.2)	50 (4.4)	< 0.001	219 (19.9)	50 (4.5)	< 0.001	915.8 (18.1)	205.0 (4.5)	< 0.001
Moderate	204 (4.6)	20 (1.7)	0.021	59 (5.3)	20 (1.8)	0.002	240.9 (4.8)	93.3 (2.1)	0.026
Severe	130 (2.9)	17 (1.5)	0.055	42 (3.8)	17 (1.5)	0.773	147.2 (2.9)	95.7 (2.1)	0.675
Secondary outcomes									
Anxiety symptoms, *n* (%)	821 (18.5)	121 (10.5)	0.001	210 (19.0)	114 (10.3)	0.001	916.6 (18.1)	462.8 (10.2)	< 0.001
Severity of anxiety symptoms, *n* (%)									
Mild	416 (9.4)	41 (3.5)	< 0.001	93 (8.4)	38 (3.4)	< 0.001	436.5 (8.6)	145.7 (3.2)	< 0.001
Moderate	237 (5.3)	42 (3.7)	0.038	87 (7.9)	41 (3.7)	0.024	307.2 (6.1)	162.9 (3.6)	0.023
Severe	168 (3.8)	38 (3.3)	0.534	30 (2.7)	35 (3.2)	0.697	172.9 (3.4)	154.2 (3.4)	0.755
Depressive or anxiety symptoms, *n* (%)	1556 (35.0)	186 (16.2)	< 0.001	401 (36.4)	179 (16.2)	< 0.001	1713.9 (33.9)	752.0 (16.6)	< 0.001
Depressive and anxiety symptoms, *n* (%)	451 (10.1)	22 (1.9)	< 0.001	129 (11.7)	22 (2.0)	< 0.001	506.6 (10.0)	104.8 (2.3)	< 0.001
Sleep disturbance, *n* (%)	2068 (46.5)	218 (19.0)	< 0.001	464 (42.1)	198 (18.0)	< 0.001	2171.5 (42.9)	793.1 (17.5)	< 0.001
Delirium, *n* (%)	528 (11.9)	94 (8.2)	< 0.001	127 (11.5)	92 (8.3)	0.015	550.1 (10.9)	383.6 (8.5)	0.048

*Note:* Data were presented as *n* (%). DEX, Dexmedetomidine; IPTW, inverse probability of treatment weighting; PSM, propensity score matching.

### 3.3 Secondary Outcomes Analysis

**FIGURE 3:**
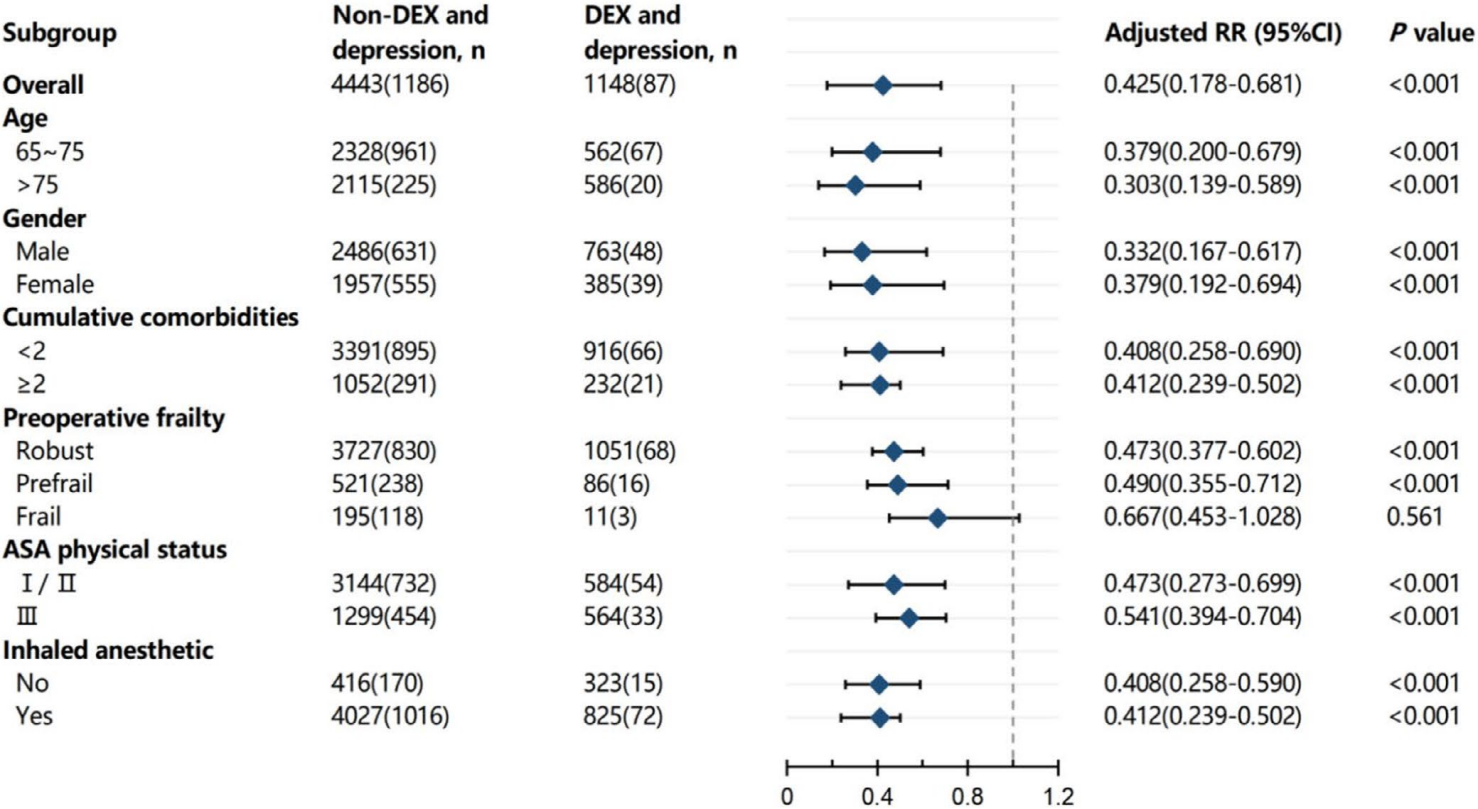
Subgroup analyses of the association between dexmedetomidine and postoperative depressive symptoms in older noncardiac surgical patients. ASA, American Society of Anesthesiologists; CI, confidence interval; DEX, Dexmedetomidine; RR, risk ratio.

In the unadjusted sample, the postoperative psychological symptoms incidences of the dexmedetomidine group were lower than those of the nondexmedetomidine group (anxiety symptom: 10.5% vs. 18.5%, *p* < 0.001; depressive symptoms or anxiety symptoms: 16.2% vs. 35.0%, *p* < 0.001; depressive symptoms and anxiety symptoms: 1.9% vs. 10.1%, *p* < 0.001, Table 2). The incidence of postoperative sleep disturbance and delirium in the dexmedetomidine group was also significantly lower than that in the nondexmedetomidine group (sleep disturbance: 19.0% vs. 46.5%, *p* < 0.001; delirium: 8.2% vs. 11.9%, *p* < 0.001). In the PSM‐adjusted sample, the postoperative psychological symptoms incidences of the DEX group were also lower than those of the nondexmedetomidine group (anxiety symptom: 10.3% vs. 19.0%, *p* < 0.001; depressive symptoms or anxiety symptoms: 16.2% vs. 36.4%, *p* < 0.001 and depressive symptoms and anxiety symptoms: 2.0% vs 11.7%, *p* < 0.001). The incidence of postoperative sleep disturbance and delirium in the dexmedetomidine group was also significantly lower than that in the nondexmedetomidine group in PSM adjustment (sleep disturbance: 18.0% vs. 42.1%, *p* < 0.001; delirium: 8.3% vs. 11.5%, *p* < 0.001). In the IPTW‐adjusted sample, similar results were obtained. The incidence of postoperative anxiety symptoms, sleep disturbance, and delirium in the dexmedetomidine group was significantly lower than that in the nondexmedetomidine group (*p* < 0.001). Whether in PSM correction or IPTW adjustment, we found that dexmedetomidine was associated with alleviating mild and moderate anxiety symptoms, but had no significant effect on severe anxiety symptoms. The use of intraoperative dexmedetomidine significantly alleviated the symptoms of anxiety symptoms and sleep disturbance in older patients who underwent urology, gynecology, gastroenterology, and thoracic surgery (Figure 3).

### 3.4 Association Between the Dexmedetomidine and Outcomes

It was noteworthy that the use of dexmedetomidine during surgery was significantly correlated with the reduction of postoperative depressive symptoms in older patients undergoing noncardiac surgery (crude random‐effect model, RR [95% CI]: 0.230 [0.180–0.295], *p* < 0.001; adjusted random‐effect model: 0.104 [0.080–0.140], *p* < 0.001; PSM model: 0.311 [0.242–0.415], *p* < 0.001; IPTW model: 0.297 [0.253–0.343], *p* < 0.001, Table 3). We found that intraoperative use of dexmedetomidine was also closely related to the reduction of postoperative anxiety symptoms in older patients undergoing noncardiac surgery (adjusted random‐effect model: 0.272 [0.224–0.356], *p* < 0.001; PSM model: 0.377 [0.283–0.487], *p* < 0.001; IPTW model: 0.533 [0.437–0.595], *p* < 0.001, Table 3). In addition, we also found that intraoperative use of dexmedetomidine was closely related to the reduction of postoperative sleep disturbances in older patients undergoing noncardiac surgery (adjusted random‐effect model: 0.283 [0.234–0.363], *p* < 0.001; PSM model: 0.668 [0.544–0.783], *p* < 0.001; IPTW model: 0.797 [0.599–0.847], *p* < 0.001, Table 3). Finally, this study demonstrated that dexmedetomidine was negatively correlated with postoperative delirium in older patients undergoing noncardiac surgery (adjusted random‐effect model: 0.784 [0.677–0.876], *p* < 0.001; PSM model: 0.684 [0.556–0.817], *p* < 0.001; IPTW model: 0.832 [0.736–0.965], *p* < 0.001, Table 3).

**TABLE 3:** Association between intraoperative dexmedetomidine use and postoperative depressive symptoms, anxiety symptoms, sleep disturbances, and delirium in older patients undergoing noncardiac surgery.

Outcome variable	Crude random‐effect model			Adjusted random‐effect model			PSM model			IPTW model		
	RR	95% CI	*p*	RR	95% CI	*p*	RR	95% CI	*p*	RR	95% CI	*p*
Primary outcome												
Depressive symptom	0.230	0.180–0.295	< 0.001	0.104	0.080–0.140	< 0.001	0.311	0.242–0.415	< 0.001	0.297	0.253–0.343	< 0.001
Secondary outcomes												
Anxiety symptom	0.534	0.431–0.661	< 0.001	0.272	0.224–0.356	< 0.001	0.377	0.283–0.487	< 0.001	0.533	0.437–0.595	< 0.001
Sleep disturbance	0.640	0.502–0.891	< 0.001	0.283	0.234–0.363	< 0.001	0.668	0.544–0.783	< 0.001	0.797	0.599–0.847	< 0.001
Delirium	0.657	0.534–0.832	< 0.001	0.784	0.677–0.876	0.009	0.684	0.556–0.817	< 0.001	0.832	0.736–0.965	0.012

*Note:* The study center was analyzed as a random effect. Nondexmedetomidine group was set as the reference (RR = 1). Variables were adjusted in all models: age, gender, MMSE score, BMI, education level, smoking and alcohol consumption, surgical history, chronic pain, history of malignancy, cumulative comorbidity, frail state, metabolic equivalent, white blood cell count, hemoglobin, ASA classification, duration of surgery, surgical grade, inhaled anesthetic drugs, NSAIDs, colloidal, transfusion, drainage and patient‐controlled analgesia, type of surgery, and follow‐up method.

Abbreviations: CI, confidence interval; IPTW, inverse probability of treatment weighting; PSM, propensity score matching; RR, risk ratio.

### 3.5 Subgroup Analyses

Based on the mixed‐effect model, we performed a subgroup analysis of postoperative depressive symptoms according to age, gender, cumulative comorbidity, frailty, ASA physical status, and intraoperative use of inhaled anesthetics (Figure 3). In the analysis of age subgroups, we found that dexmedetomidine was associated with reducing the incidences of postoperative depressive symptoms in both 65 ~ 75 years and > 75 years subgroups (65 ~ 75 years RR [95% CI]: 0.379 [0.167–0.617], *p* < 0.001; > 75 years:0.303 [0.139–0.589] *p* < 0.001). Dexmedetomidine had a protective effect on postoperative depressive symptoms in older patients with noncardiac surgery in both men and women (male: 0.332 [0.167–0.617], *p* < 0.001; female: 0.379, [0.192–0.694], *p* < 0.001). At the same time, dexmedetomidine in different cumulative comorbidity subgroups (< 2 and ≥ 2), ASA physical status (I/II and III), and inhaled anesthetics subgroups (no and yes) had a protective effect on postoperative depressive symptoms in older patients undergoing noncardiac surgery (*p* < 0.001). However, dexmedetomidine was negatively correlated with postoperative depressive symptoms only in the robust patients and the prefrail subgroup (robust: 0.473 [0.377–0.602], *p* < 0.001; prefrail: 0.490 [0.355–0.712], *p* < 0.001), and there was no significant difference in the frail subgroup (*p* = 0.561, Figure 3).

## 4 Discussion

Using prospective longitudinal observation data from nine general hospitals in China, this research explored the relationship between intraoperative dexmedetomidine and neuropsychiatric disorders within 7 days after operation in older patients undergoing noncardiac surgery. It was worth noting that we found that intraoperative dexmedetomidine was significantly associated with reducing postoperative depressive symptoms, anxiety symptoms, sleep disturbances, and delirium in older patients. The results of this study confirmed its effectiveness and robustness by propensity matching, inverse probability weighting method, random‐effect model, and subgroup analysis. Therefore, the intraoperative use of dexmedetomidine may provide a new perspective and direction for alleviating postoperative neuropsychiatric disorders in older patients.

Acknowledging the importance of older patients' neuropsychiatric well‐being was crucial in mitigating the healthcare burden [29]. This study focuses on the emergence of postoperative psychological symptoms, indicating that these conditions arise during hospitalization, particularly due to surgery, rather than existing before This contrasts with patients who already exhibit anxiety symptoms and depressive symptoms before surgery. Investigating the development of new postoperative psychological symptoms could elucidate the causal link between surgery and patient mental health, uncover factors that might precipitate or intensify mental health issues during the surgical process, and furnish more precise suggestions for enhancing patient care. Moreover, this study can furnish guidance for medical professionals in managing newly diagnosed postoperative mental health issues, enabling doctors to better comprehend patient requirements and formulate more tailored treatment strategies [30].

In this study, we found that the prevalence of postoperative depressive symptoms in older patients who underwent noncardiac surgery ranged from 20% to 35%, and anxiety symptoms from 10% to 20%, findings in line with prior research [31]. The study found that the incidence of postoperative depressive symptoms was 34% in patients undergoing gastrointestinal surgeries, 32% in patients undergoing gynecological surgeries, and 30% in elderly patients undergoing ear, nose, and throat surgeries. In addition, we found that the short‐term depressive symptoms of dexmedetomidine patients were significantly lower than those in the nondexmedetomidine group by comparing all seven noncardiac surgeries, which has important clinical significance and broad applicability. This study also reported a 40% incidence of postoperative anxiety symptoms after urological surgery, 36% after gynecological surgery, and 27% in elderly patients undergoing gastrointestinal surgery. Interestingly, the order of high and low rates of postoperative anxiety symptoms and depressive symptoms is not consistent. Different types of surgery have different physical and psychological effects on patients. However, the similarity is that the occurrence of postoperative depressive symptoms and anxiety symptoms in dexmedetomidine patients was significantly lower than that in the nondexmedetomidine group.

The investigation of therapeutic strategies for postoperative depressive symptoms in surgical patients continues to be a prominent area of research [32, 33]. Ketamine, as a novel antidepressant, offers new therapeutic possibilities for depressive patients, albeit with concerns over its diverse side effects and potential for addiction [34]. Prior to its recognition for antidepressant properties, ketamine served as an anesthetic, thereby suggesting avenues for exploring anesthetics' potential in mitigating perioperative mental disturbance. Surgical factors, including trauma, tissue injury, and the activation of immune cells, can stimulate the immune system and provoke inflammatory responses [35]. Dexmedetomidine exhibits immunomodulatory effects, contributing to the preservation of immune equilibrium within the body. Concurrently, dexmedetomidine can suppress excessive inflammatory responses and decrease postoperative inflammation, which is associated with a lower incidence of complications in patients postsurgery. Numerous studies have established a robust link between heightened inflammatory biomarkers and the risk of mental disorders, with this causal relationship consistently substantiated in preclinical research [36]. Moreover, dexmedetomidine can modulate γ‐aminobutyric acid (GABA) receptors, facilitating the reduction of neural excitability and thereby alleviating anxiety symptoms and tension [37]. These therapeutic effects also appear advantageous for older patients.

In our study, 66.0% of older patients underwent major surgery (Level 4), with 34.1% classified as ASA class III, and 40.3% experiencing surgery lasting 3 h or more. Major surgery is associated with significant pain and could severely impact sleep quality. We observed a high prevalence of postoperative sleep disturbance in older patients following major noncardiac surgery, reaching 30%–41%. Our analysis revealed a negative correlation between the use of dexmedetomidine in noncardiac surgery and the incidence of postoperative sleep disturbance. Studies have indicated that dexmedetomidine can enhance deep sleep, enhance sleep quality, and decrease nighttime awakenings, contributing to a consistent sleep state throughout the night [38]. Dexmedetomidine exerts its effects on the central nervous system through α adrenergic receptors, which induce sedative and calming effects. This sedative effect aids in alleviating anxiety symptoms and tension, which in turn facilitates improved sleep onset and enhanced sleep quality [39, 40]. This may account for one of the factors contributing to improved sleep quality and a decreased incidence of sleep disturbance. Nonetheless, the precise pharmacological mechanism remains elusive, and further investigation is required to elucidate it.

We found that the postoperative delirium incidence among older patients undergoing noncardiac surgery was found to be between 8% and 12%, aligning with earlier research reporting a range of 6%–11% [12, 13]. Notably, our research revealed a negative correlation between intraoperative administration of dexmedetomidine and the development of postoperative psychological symptoms in older surgical patients who did not require ICU admission. Consequently, intraoperative dexmedetomidine has been demonstrated to correlate with postoperative delirium in both ICU and non‐ICU patients, offering crucial scientific guidance for the broad application of dexmedetomidine in clinical settings aimed at delirium prevention.

We also found that dexmedetomidine remained the protective factor for postoperative depressive symptoms in subgroups of patients with different ages, genders, cumulative comorbidity, and ASA grades. By identifying and understanding this protective factor, doctors can take corresponding intervention measures to increase patient protection and reduce the incidence of adverse outcomes. It should be noted that subgroup analysis only observes the correlation between different subgroups and cannot determine causal relationships. Researchers may further explore the mechanism and specific ways of action of this factor to deepen their understanding of its role and provide a basis for developing more effective intervention measures.

There are several limitations. First, this analysis included only older surgical patients from China, and some intraoperative management strategies for our patients may differ from those used in other countries. Secondly, our study did not encompass patients who were admitted to the ICU postoperatively. Previous research has indicated that the ICU experience can be a significant psychological trauma, potentially leading to cumulative effects on postoperative psychological symptoms. Additionally, the use of low‐dose dexmedetomidine in the ICU could introduce bias in the results. This subset of patients presents an opportunity for future research. Thirdly, the data collected were observational and did not specify the dosage of dexmedetomidine, making it impossible to determine the optimal dosage for use. Fourthly, we did not conduct a longer‐term follow‐up after surgery. While we recognize that 7 days postoperatively is typically considered a critical period for transitioning from surgical anesthesia to recovery, we did not explore the longer‐term psychological recovery in older patients. Furthermore, the long‐term neuropsychological effects of continuous dexmedetomidine administration during surgery on postoperative patients remain unclear. Consequently, our team is undertaking a multicenter randomized controlled trial to investigate the relationship between dexmedetomidine and postoperative neuropsychiatric disorders in a more comprehensive and in‐depth manner.

## 5 Conclusion

Intraoperative dexmedetomidine was associated with a reduction in the incidence of postoperative 7‐day depressive symptoms, anxiety symptoms, sleep disturbances, and delirium. Dexmedetomidine may offer innovative strategies and directions for the management of postoperative neuropsychiatric symptoms in older surgical patients. Consequently, additional randomized controlled trials are warranted to confirm the postoperative neuropsychiatric symptoms’ therapeutic value of dexmedetomidine in older patients undergoing noncardiac surgery.

## Author Contributions

Xinyu Hao: investigation, formal analysis, methodology, and project administration. Zhuoning Zhang: investigation and resources. Lujia Yang: formal analysis, methodology, visualization, and writing – original draft. Yongxin Guo: investigation, formal analysis, and funding acquisition. Fuyang Cao: investigation and formal analysis. Jiangbei Cao: investigation and resources. Yanhong Liu: investigation and resources. Jingsheng Lou: investigation and resources. Ziyao Xu: investigation and resources. Zhuoning Zhang: investigation and resources. Yulong Cui: resources. Yunxiao Bai: resources. Xiaoping Gu: resources. Difen Wang: resources. Qianyu Cui: resources. Zhikang Zhou: resources. Hao Shen: resources. Jingjia Sun: resources. Weidong Mi: conceptualization, data curation, funding acquisition, validation, and writing – review and editing. Li Tong: conceptualization, data curation, methodology, funding acquisition, supervision, and writing – review and editing.

## Conflicts of Interest

The authors declare no conflicts of interest.

## Supporting information


**Figure S1.** Use rate of dexmedetomidine in seven noncardiac surgery departments.
**Figure S2.** Kernel density before and after match. (A) K‐density curves before match. A total of 4443 patients were in nondexmedetomidine group and 1148 patients in dexmedetomidine group. (B) K‐density curves after propensity scores match. A total of 1103 patients of nondexmedetomidine and 1103 patients of dexmedetomidine. (C) K‐density curves after inverse probability of treatment weighting.
**Figure S3.** Incidence of postoperative depressive symptoms, anxiety symptoms, sleep disturbance, and delirium in older patients in seven different surgical departments. (A) The incidence of postoperative depressive symptoms with or without dexmedetomidine in different departments. (B) The incidence of postoperative anxiety symptoms with or without dexmedetomidine in different departments. (C) The incidence of postoperative sleep disturbance with or without dexmedetomidine in different departments. (D) The incidence of postoperative delirium with or without dexmedetomidine in different departments. *p* < 0.05 showed as ✱. *p* < 0.01 showed as ✱✱. *p* < 0.001 showed as ✱✱✱.

## Data Availability

The data that support the findings of this study are available from the corresponding author upon reasonable request.
