# Supplementary material for: Association of Dexmedetomidine With Postoperative Depressive Symptoms in Older Surgical Patients: A Prospective Multicenter Study
Source: CNS Neurosci Ther. 2025 May 19;31(5):e70407. doi: 10.1111/cns.70407 (PMC12087293; doi:10.1111/cns.70407)
Supplement: Supplementary file 1 — Figure S1. Use rate of dexmedetomidine in seven noncardiac surgery departments. Figure S2. Kernel density before and after match. (A) K‐density curves before match. A total of 4443 patients were in nondexmedetomidine group and 1148 patients in dexmedetomidine group. (B) K‐density curves after propensity scores match. A total of 1103 patients of nondexmedetomidine and 1103 patients of dexmedetomidine. (C) K‐density curves after inverse probability of treatment weighting. Figure S3. Incidence of postoperative depressive symptoms, anxiety symptoms, sleep disturbance, and delirium in older patients in seven different surgical departments. (A) The incidence of postoperative depressive symptoms with or without dexmedetomidine in different departments. (B) The incidence of postoperative anxiety symptoms with or without dexmedetomidine in different departments. (C) The incidence of postoperative sleep disturbance with or without dexmedetomidine in different departments. (D) The incidence of postoperative delirium with or without dexmedetomidine in different departments. p < 0.05 showed as ✱. p < 0.01 showed as ✱✱. p < 0.001 showed as ✱✱✱. [file CNS-31-e70407-s001.docx]

**Supplementary Appendix**

Dexmedetomidine use rate in 7 non-cardiac surgeries


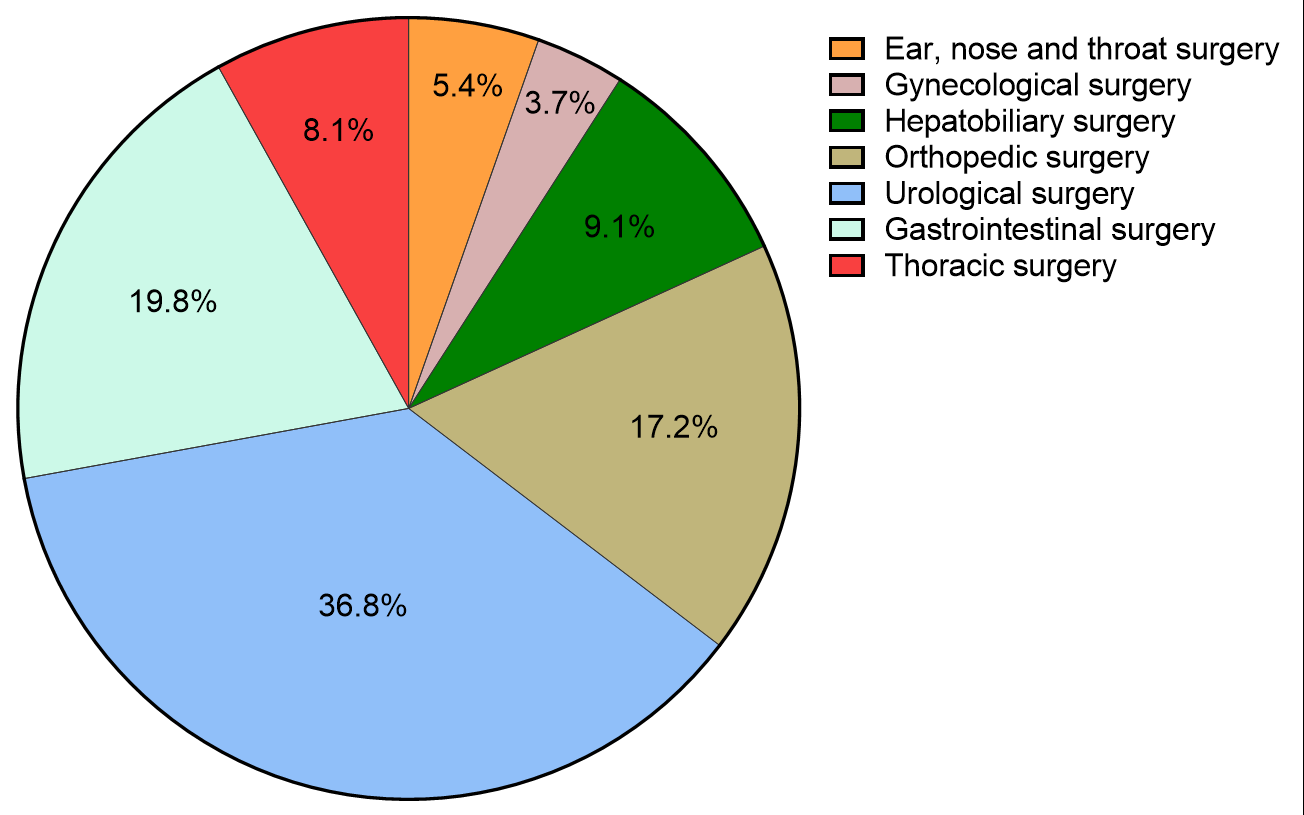


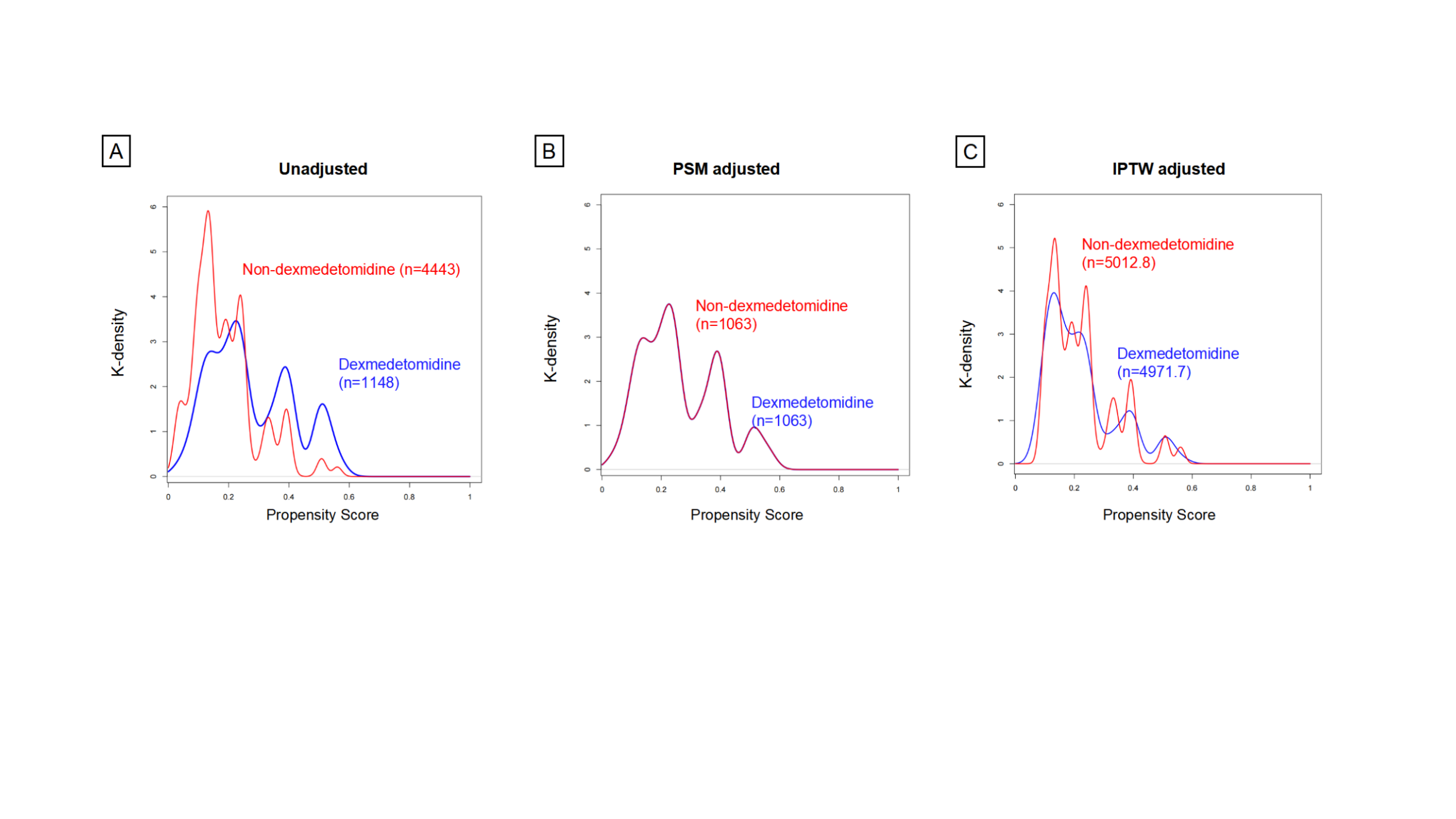


**
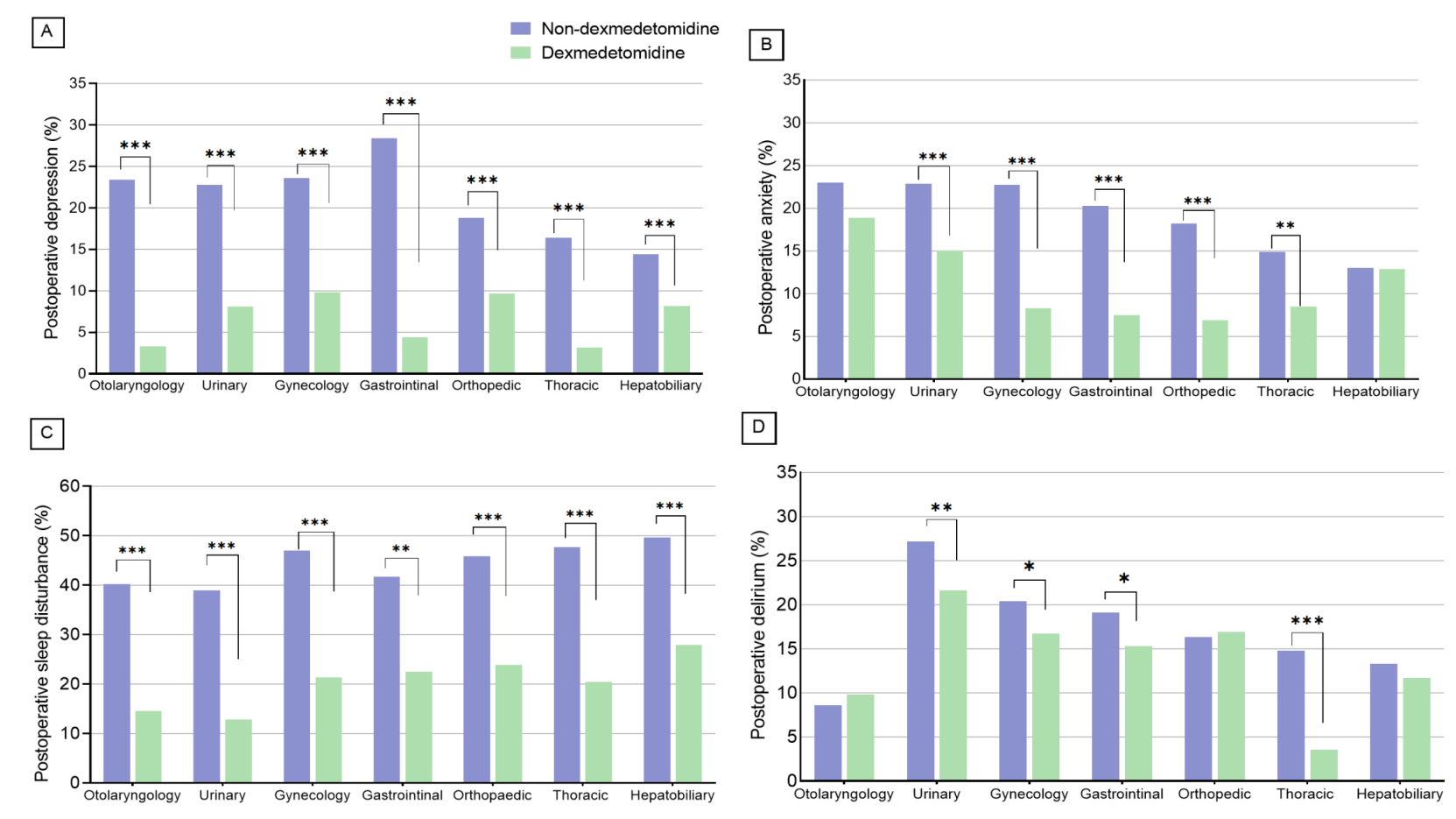
**

**Association of Dexmedetomidine with Postoperative Depressive Symptom in Older Surgical Patients: A Prospective Multicenter Study**
